# Effect of nanocellulose polymorphism on electrochemical analytical performance in hybrid nanocomposites with non-oxidized single-walled carbon nanotubes

**DOI:** 10.1007/s00604-021-05161-w

**Published:** 2022-01-14

**Authors:** Silvia Dortez, Tania Sierra, Miguel Á. Álvarez-Sánchez, José M. González-Domínguez, Ana M. Benito, Wolfgang K. Maser, Agustín G. Crevillen, Alberto Escarpa

**Affiliations:** 1 Department of Analytical Chemistry, Physical Chemistry and Chemical Engineering, University of Alcalá, 28871 Alcalá de Henares, Madrid Spain; 2 Group of Carbon Nanostructures and Nanotechnology, Instituto de Carboquímica, ICB-CSIC, C/ Miguel Luesma, Castán 4, 50018 Zaragoza, Spain; 3 Department of Analytical Sciences, Faculty of Sciences, Universidad Nacional de Educación a Distancia (UNED), 28040 Madrid, Spain; 4 Chemical Research Institute “Andrés M. del Río” (IQAR), University of Alcalá, 28805 Alcalá de Henares, Madrid Spain

**Keywords:** Single-walled carbon nanotube, Green chemistry, Cellulose nanocrystal, Sustainability, Electrochemical detection

## Abstract

Two cellulose nanocrystals/single-walled carbon nanotube (CNC/SW) hybrids, using two cellulose polymorphs, were evaluated as electrochemical transducers: CNC type I (CNC-I/SW) and CNC type II (CNC-II/SW). They were synthesized and fully characterized, and their analytical performance as electrochemical sensors was carefully studied. In comparison with SWCNT-based and screen-printed carbon electrodes, CNC/SW sensors showed superior electroanalytical performance in terms of sensitivity and selectivity, not only in the detection of small metabolites (uric acid, dopamine, and tyrosine) but also in the detection of complex glycoproteins (alpha-1-acid glycoprotein (AGP)). More importantly, CNC-II/SW exhibited 20 times higher sensitivity than CNC-I/SW for AGP determination, yielding a LOD of 7 mg L^−1^.These results demonstrate the critical role played by nanocellulose polymorphism in the electrochemical performance of CNC/SW hybrid materials, opening new directions in the electrochemical sensing of these complex molecules. In general, these high-active-surface hybrids smartly exploited the preserved non-oxidized SW conductivity with the high aqueous dispersibility of the CNC, avoiding the use of organic solvents or the incorporation of toxic surfactants during their processing, making the CNC/SW hybrids promising nanomaterials for electrochemical detection following greener approaches.

**Graphical abstract:**

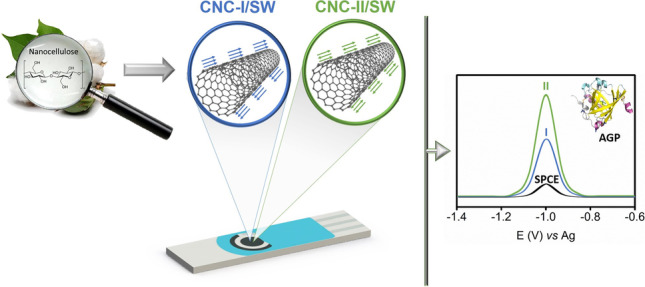

**Supplementary Information:**

The online version contains supplementary material available at 10.1007/s00604-021-05161-w.

## Introduction

The need for sustainable alternatives to conventional materials has led to a growth in demand for bio-based materials directed to applications in different areas of the industry. Among them, nanocellulose (NC) has become a promising alternative in nanotechnology applications and the focus of intensive research, because of its attractive properties such as biocompatibility, biodegradability, renewable nature, non-toxicity, anisotropic shape, excellent mechanical properties, tailorable surface chemistry, and interesting optical properties [1–4].

Nanocellulose can be categorized into three types: (I) cellulose nanocrystals (CNC), also referred to as nanocrystalline cellulose; (II) cellulose nanofibrils, also referred to as nano-fibrillated cellulose; and (III) bacterial cellulose [1, 4]. Besides, CNC shows at least four main polymorphs, namely cellulose I, cellulose II, cellulose III, and cellulose IV, being I and II differentiated upon the different orientations of cellulose chains [5]. Cellulose I and II are the most studied and reported [6]. Briefly, type I is dominant in natural cellulose sources and exhibits a parallel arrangement of chains with sharp and straight needle-like morphology, while type II is obtained by dissolution and recrystallization of type I, displaying an antiparallel arrangement with shorter and twisted rod-like nanocrystals [5]. These differences between types I and II in their structures and morphologies provide them with different properties and behavior. For example, CNC type II is more enzymatically degradable than type I [7], and also, these nanostructures may exert very different properties to their hybrids with other nanostructures. As a matter of example, we recently demonstrated that dispersions of SWCNT with CNC type II exhibited selective cytotoxicity on human colon cancer (Caco-2) cells, with no harm on equivalent normal (healthy) cells, while the SWCNT hybrids with CNC type I were generally innocuous [6].

Taking advantage of the aforementioned features, NC has been extensively applied in drug delivery [8], environmental monitoring and remediation [9, 10], food safety [11, 12], and chemical/physical/mechanical sensing [13, 14]. Regarding electrochemical sensors, the interest in NC is recent. As NC is not conductive, a general strategy to circumvent this issue is to combine it with carbon nanomaterials (CNM, such as carbon nanotubes (CNT) or graphene derivatives) designing hybrid materials with high conductivity and enhanced mechanical strength [15–19]. The preference for this synergistic approach is because CNM and NC have high affinity for each other. Furthermore, the favorable interactions between CNM and NC prevent their natural tendency to self-aggregate [20]. As a matter of fact, some authors have identified such favorable interaction between CNT and CNC as the association of hydrophobic (200) planes of CNC with the (also very) hydrophobic sp^2^ carbon lattice of CNT, entailing the alignment of CNC along the tube axis in SWCNT [21].

Another advantage of these hybrids is that they are readily dispersed in water while pristine or poorly oxidized CNM are difficult to disperse in water and organic solvents [1]. This is very interesting in sensor fabrication because it allows preparing it without using organic solvents, approaching to the Green Chemistry concept [22, 23]. Moreover, these NC/CNM hybrids can be used to produce electrically conductive fabric for developing high-performance wearable sensors [24].

There are only a few examples in the literature about the use of NC/CNM hybrids for electrochemical sensing despite the potential advantages [15–19]. Shahrokhian’s group reported a cellulose nanofiber/nanocarbon modified electrode for the detection of two drugs: clonazepam [19] and metoclopramide [18]. An increase in the electroactive surface area of sensors and in the analyte peak currents was demonstrated by using this hybrid material. However, the type and characteristics of the used nanocellulose were not reported. Shalauddin *et al.* developed a hybrid NC/functionalized MWCNT nanocomposite for the electrochemical sensing of diclofenac sodium in pharmaceutical drugs and biological fluids [15]. MWCNTs were functionalized with carboxylic groups by strong acid treatment. This hybrid nanocomposite showed lower electron transfer resistance and better sensitivity towards diclofenac than each nanomaterial separately. Ortolani *et al.* prepared a glassy carbon electrode modified by single-walled carbon nanohorns and NC for sensing of adenine and guanine [16]. This NC/single-walled carbon nanohorn hybrid provided a strong electrocatalytic response towards both analytes, increasing the sensor sensitivity. Finally, a silylated graphene oxide–grafted-chemically modified nanocellulose (Si-GO-g-CMNC) was fabricated for electrochemical sensing of cholesterol [17]. This new material was integrated in a molecular imprinting polymer sensor to carry out a selective detection of cholesterol.

However, in the last three references [15–17], the authors used the term “nanocellulose” in a generic way and did not mention or study the type of nanocellulose polymorph (I–IV) being employed. Nevertheless, according to X-ray diffraction (XRD) pattern reported in these articles, CNC were of type I that is the natural polymorph of cellulose. In fact, very scarce works in NC/CNM type II hybrids have been reported to date [6].

Regarding the different properties reported in the literature for CNC types I and II [7], and their respective CNM hybrids [6], it seems pertinent to study each nanocellulose polymorph (i.e., crystalline structure) in the electrochemical performance of NC/CNM hybrid sensors, because unique or novel responses and performances could arise. Additionally, the use of non-oxidized CNM is of relevance to preserve their conductive properties, but it is scarcely addressed, despite the excellent dispersive action of CNC towards non-oxidized CNTs in water [6, 21].

In consequence, we synthesized two different non-oxidized CNC/SW hybrids: one using CNC type I (CNC-I/SW) and another using CNC type II (CNC-II/SW). They were fully characterized, and their electrochemical performance was carefully studied and compared to bare SWCNTs and screen-printed carbon electrodes (SPCEs). Then, CNC/SW hybrids were used as transducers in the development of electrochemical sensors for detecting target compounds with clinical interest (uric acid, dopamine, tyrosine, and alpha-1-acid glycoprotein) by differential pulse voltammetry (DPV). The aim of the present work is to shed light on the potential interest of CNC hybrids with CNM as regards electrochemical sensing technologies, and the relevance that the CNC polymorphism acquires in such scenario.

## Materials and methods

### Reactive and materials

K_4_Fe(CN)_6_·3H_2_O, K_3_Fe(CN)_6_, Ru(NH_3_)_6_Cl_3_, *N*,*N*-dimethylformamide (DMF), alpha-1-acid glycoprotein (AGP) (≥ 99%), transferrin (≥ 98%), human serum (certified reference material, ERM-DA470K/IFCC), potassium osmate (VI) dihydrate, *N*,*N*,*N*′,*N*′-tetramethylenediamine (TEMED), potassium nitrate (≥ 98%), uric acid, dopamine, l-tyrosine, and cellulose microcrystalline powder (20 µm) were purchased from Sigma–Aldrich (Darmstadt, Germany). Hydrochloric acid (37%), sulfuric acid (95–98%), sodium chloride, disodium hydrogen phosphate, and sodium dihydrogen phosphate were purchased from PanReac (Barcelona, Spain).

SWCNTs were acquired from Carbon Solutions Inc. (type P2-SWNT, Riverside, CA, USA). These correspond to a purified material following a non-specified non-oxidative process.

For the synthesis of CNC and characterization of CNC/SW hybrids, ultrapure water was obtained from a Siemens Ultraclear device, with a conductivity of 0.055 μS cm^−1^. For the analytical studies, all aqueous solutions were prepared in Milli-Q water (Merck Millipore, Darmstadt, Germany).

### Instrumentation

A Shimadzu UV-2401PC spectrophotometer was used to determine the SWCNT concentration in both dispersed hybrids, which were diluted with ultrapure water to adjust the absorbance to ~ 0.4 at 850 nm, using ultrapure water for blank measurements.

Near infrared (NIR) absorption spectroscopy was performed in the liquid phase using a Bruker VERTEX 70 spectrometer and 2 mL polystyrene cuvettes using the same dilution as that of UV–Vis spectroscopy.

Particle size and *ζ*-potential were measured from the diluted dispersions (absorbance ~ 0.4 at 850 nm) in a Malvern Nano ZS instrument, according to the principles of dynamic light scattering (DLS) and electrophoresis, respectively. The device measures the particle size distribution through the DLS approach by irradiating with a He − Ne laser at 633 nm and applying the Stokes − Einstein equation while assuming a single and constant diffusion rate (i.e., spherical particles). The *ζ*-potential values were calculated from the electrophoretic mobility using Henry’s equation. For the DLS measurements, a refractive index of 2.42 (carbon) was taken for SWCNT hybrids. Measurements were carried out in disposable polystyrene dip cells (code DTS1061). All *ζ*-potential measurements were performed at least in triplicates and are referred to an average pH of 5.2 and a room temperature of 25 °C.

XRD patterns were collected with a Bruker D8 Advance diffractometer using a Cu tube as the X-ray source (*λ*_CuKα_ = 1.54 Å), a tube voltage of 40 kV, and a current of 40 mA. The results were recorded in Bragg − Brentano geometry in the range of 2*θ* = [5 − 40°], with steps of 0.05° and 3 s accumulation time. Samples were measured in the form of spongy freeze-dried solids from the corresponding aqueous dispersions of CNC/SW hybrids and analyzed with the fitting software Topas 5.0.

The textural characterization of CNC/SW freeze-dried hybrids was carried out by N_2_ physisorption at 77 K in a Micromeritics ASAP2020 device. The specific surface area (*S*_BET_) was calculated by applying the Brunauer–Emmett–Teller (BET) method from the data obtained from the isotherms. The total pore volume (*V*_T_) was obtained from the adsorbed N_2_ amount at a relative pressure of *P*/*P*_0_ = 0.98.

Scanning electron microscopy (SEM) was undertaken to characterize the CNC/SW morphology, by use of a JSM-IT500 (Jeol, Japan), under an accelerating voltage of 25 kV. Images were taken in the secondary electron mode.

Potentiostat Autolab PGSTAT204 (Metrohm-Autolab, Utrecht, The Netherlands) was used for all electrochemical and impedance measurements. This instrument was controlled by the software Nova 1.10.

SPCEs (DRP-110, DropSens, Oviedo, Spain) were employed. They consist of carbon working electrode (4 mm diameter), carbon counter electrode, and silver reference electrode. This kind of SPCEs works as an electrochemical cell, which needs a minimum volume of 50 μL.

### Procedures

#### Synthesis of CNC/SW type I and II

Synthesis of both NC types I and II was performed according to the process described by a preceding work in our laboratories, reported elsewhere [6] (see also Supplementary Information (SI)).

For the preparation of CNC/SW hybrids [6], in a typical experiment, 20 mg of SWCNTs was added to 20 mL of the CNC aqueous colloid (2.5 mg mL^−1^), either type I or II, and sonicated using a sonic tip (Hielscher DRH-P400S; 400 W maximum power; 24 kHz maximum frequency at 60% amplitude, and 50% cycle time) for 1 h while cooling with an external ice bath. The obtained dispersions were then centrifuged at 1842 rcf for 4 min in Falcon tubes (20 mL). The supernatant liquids were collected, and the sediment pellets were discarded.

Aliquots of both hybrid aqueous dispersions were freeze-dried to determine the SWCNT content in the sample, taking as a reference the SWCNT concentration value previously determined from UV–Vis-NIR spectroscopic measurements [25]. The absorbance value was taken at 850 nm to avoid influences from the background absorption, and possible changes in the intensity of SWCNT absorption bands [25]. The absorption coefficient (*ε*_850_= 27.0 mL cm^−1^ mg^−1^) was calculated applying the Lambert–Beer law: *A*_λ_ =  *ε*·*l·*c, where *A*_λ_ is the absorbance at a specific wavelength excitation (850 nm in this case), *l* is the optical pathway given by the cuvette length, and *c* is the concentration of SWCNTs expressed on mg mL^−1^. The CNC-I/SW and CNC-II/SW dispersions had a SWCNT concentration of 0.75 and 0.74 mg mL^−1^, respectively.

#### Electrode fabrication

A control SWCNT suspension was prepared adding 13 mg of as-received SWCNT in 50 mL of DMF. Then, this suspension was sonicated for 1 h using an ultrasound bath (3,000,683, P. Selecta, Barcelona, Spain) and an ultrasound probe (VCX130, Sonics, Newtown, USA) for 15 min.

Stock aqueous dispersions of CNC-I/SW and CNC-II/SW were adequately diluted to obtain a final SWCNT concentration of 0.26 mg mL^−1^ for electrode preparation.

Three kinds of electrodes were built: one modified with as-received SWCNT (SWCNT-SPE), another modified with CNC-I/SW, and, finally, another modified with CNC-II/SW. Drop casting technique was used for the fabrication of all electrodes. A total of 10 µL of CNC-I/SW and CNC-II/SW aqueous dispersions and SWCNT suspension (0.26 mg mL^−1^ SWCNT material concentration) were dropped on the surface of the ceramic slab corresponding to the former working electrode of SPCE (carbon working electrode was previously removed with a scalpel) and allowed them to dry at room temperature. Then, silver paint was used as electrical contact with the working electrode. Finally, this electric contact was isolated by using nail polish. This methodology assures us that electrochemical transduction is exclusively due to the SWCNTs.

#### Electrochemical measurements

Each type of electrode was characterized by electrochemical impedance spectroscopy (EIS) (frequencies from 100,000 to 0.01 Hz) and by cyclic voltammetry (CV) (scan rate 100 mV s^−1^) using 5 mM K_4_Fe(CN)_6_/K_3_Fe(CN)_6_ in 0.1 M KCl electrolyte as redox probe.

The analytes were detected by DPV with a pulse amplitude of 70 mV and at a scan rate of 26 mV s^−1^ (anodic sweeping). All measurements were performed using phosphate buffer saline (PBS) at pH 7.4, except measurements of AGP that were performed using phosphate buffer (PB) at pH 7.0.

AGP was labeled with the electrochemical tag Os(VI)O_2_(OH)_2_TEMED complex (AGP-Os(VI) adduct), following the previously reported protocol [26, 27].

For the analysis of serum samples, AGP contained in the sample was firstly labeled with the electrochemical tag according to the aforementioned protocol. Briefly, 200.5 μL of serum sample was mixed with 49.5 μL of Os(VI)O_2_(OH)_2_TEMED solution and it was shaken for 16 h at 37 °C and 950 rpm. Next, Amicon filters (cutoff 10 kDa) were used for removing the excess of reagent and potential interferents such as small carbohydrates labeled with Os(VI) and other small compounds present in serum. AGP-Os(VI) adduct was retained in the filter. Then, this adduct was isolated from the rest of proteins by a selective acidic precipitation [28]. Briefly, 50 μL of the previous solution and 100 μL of 0.5 M perchloric acid were vortex mixed in a microtube for 20 s. Then, the acidified solution was centrifuged (Microcentrifuge MiniSpin® Eppendorf, Germany; 3000 g for 20 min). Finally, the supernatant was neutralized by adding 0.5 M sodium hydroxide solution, and then properly diluted in 50 mM PB pH = 7.0 for DPV analysis.

All experiments were performed at room temperature.

## Results and discussion

### Structural characterization of CNC/SW hybrids

The freshly prepared CNC and CNC/SW dispersions were evaluated by DLS and electrophoresis. The determined size, polydispersity index (PDI), and electrostatic stability are shown in Table 1. The size is expressed in terms of apparent radius, meaning the hydrodynamic radius. For CNC aqueous colloids, the apparent size of CNC type II is observed as the double of CNC type I, seemingly due to the interaction of CNC type II with a higher number of water molecules, spawned by their higher amount of surface sulfate ester groups [6], leading to an increased hydrodynamic size. For CNC/SW hybrids in water suspensions, the apparent size value is highly reduced if compared to the apparent CNC radius, and still higher for CNC-II/SW hybrid. The decrease of size of CNC/SW hybrids, compared to the preceding CNC colloids, is arguably given by an effective SWCNT debundling and individualization, as previously reported [6].

**Table 1:** Hydrodynamic (apparent) size, PDI, *ζ*-potential, and NIR purity index of CNC/SW dispersions

Dispersion	Apparent radius (nm)	PDI	ζ-potential (mV)	SWCNT concentration (mg mL^−1^)	NIR purity index
CNC-I	115 ± 5	0.507 ± 0.007	− 40.8 ± 0.5	-	-
CNC-II	344 ± 8	0.44 ± 0.02	− 32.2 ± 0.9	-	-
CNC-I/SW	98 ± 1	0.265 ± 0.006	− 29.4 ± 0.3	0.75	0.16
CNC-II/SW	231 ± 1	0.47 ± 0.01	− 30.7 ± 0.4	0.74	0.15

Electrophoresis measurements provide the *ζ*-potential value, directly related to the electrostatic-based stability. CNC and CNC/SW hybrids in dispersion exhibited values in the range of − 30 to − 45 mV, being the negative sign consistent with the CNC native surface charge. An aqueous dispersion may be considered stable above 20 mV in absolute value, but this parameter would only account for the electrostatic components to the overall stabilization.

Vis–NIR absorption is one of the most accurate techniques to evaluate SWCNT purity in liquid dispersion (see further discussion in SI). Visible-NIR spectra (see SI, Fig. S1) show the peaks corresponding to electronic transitions of metallic (M_11_) and semiconducting (S_33_ and S_22_) SWCNTs. The observed features are consistent with SWCNTs dispersed with commercial surfactants, such as SDBS or SDS [25]. The NIR purity index (Table 1) was obtained by dividing the S_22_ neat band area by the whole spectral area in the same region (850–1200 nm). We chose this specific transition for being the most prominent in this kind of SWCNTs. This ratio accounts for the carbonaceous purity of a SWCNT sample (SWCNT vs other carbon forms). In general terms, there is a visible purification effect by dispersing and centrifuging SWCNTs in CNC aqueous colloids.

**Fig. 1:**
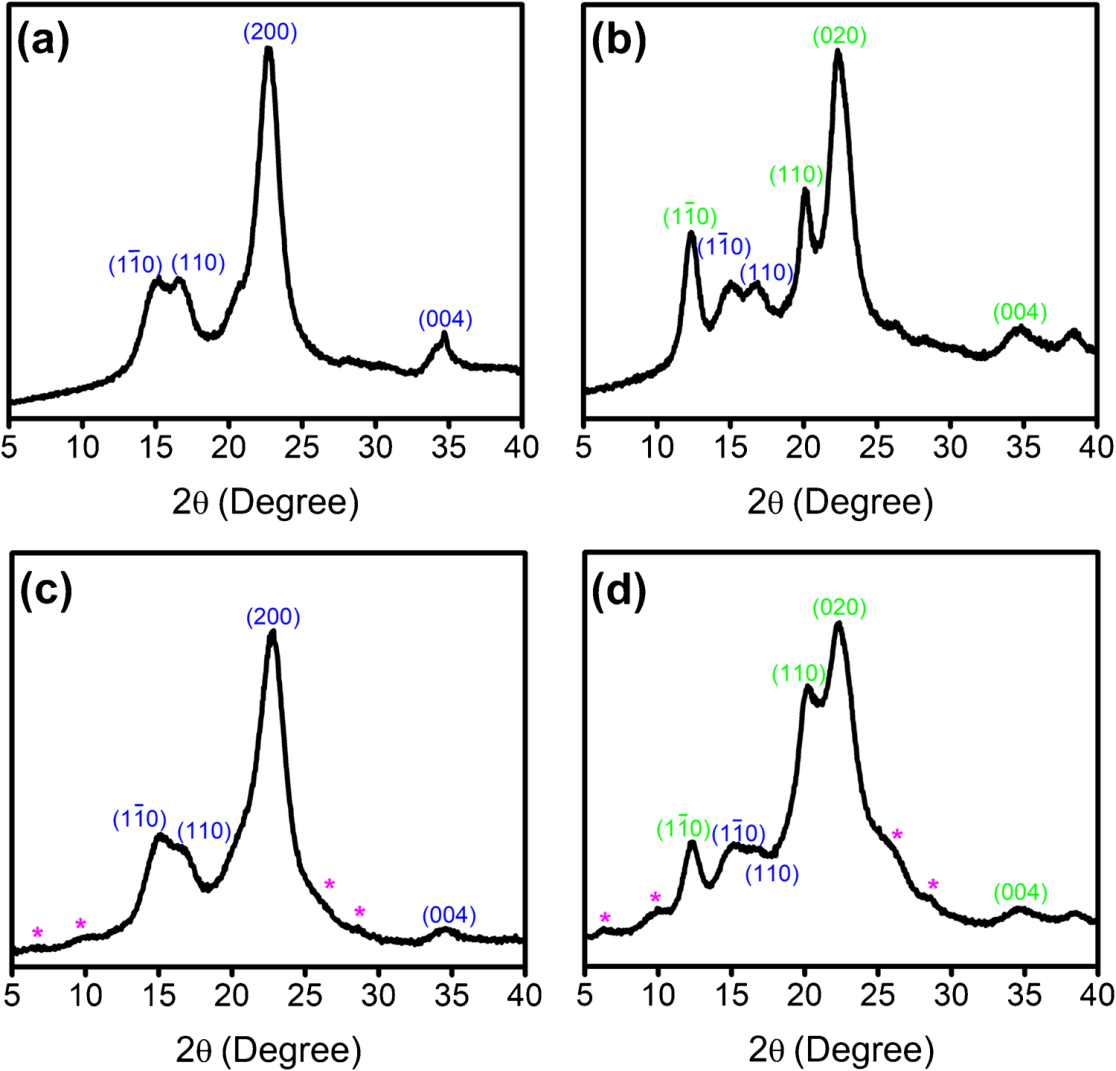
XRD patterns of freeze-dried **a** CNC types I and **b** II, together with freeze-dried dispersions of **c** CNC-I/SW and **d** CNC-II/SW, with the associated crystalline planes of CNC type I (blue) and CNC type II (green). Asterisks denote the SWCNTs’ original features

Once the characterization of CNC/SW hybrids in aqueous medium has been performed, these dispersions were freeze-dried as specified in the experimental section, yielding a spongy solid. According to weight measurements before and after drying, and taking the SWCNT concentration value as a reference, we estimated a SWCNT content within the dried solid of 42 wt% and 36 wt% (for CNC-I/SW and CNC-II/SW, respectively). These solids were characterized by XRD, to evaluate the crystalline structure of NC allomorphs in each hybrid (Fig. 1). We could not notice any crystalline feature coming from SWCNTs, mostly ascribed to the efficient debundling and individualization, coupled to the fact that the freeze-dried solids contained a much larger mass proportion of CNC. We found the four characteristic peaks of CNC type I (Fig. 1a) at 2*θ* = 14.9°, 16.6°, 22.7°, and 34.6°, corresponding to the (11̅0), (110), (200), and (004) crystalline planes, respectively. In the case of the CNC type II hybrid (Fig. 1b), the peaks observed are placed at 2*θ* = 12.4°, 20.1°, 22.2°, and 34.7°, corresponding to the crystalline planes (11̅0), (110), (020), and (004), respectively, and typically found in CNC type II structures. Additionally, the CNC type II hybrid pattern shows two minor peaks at 2*θ* = 14.8° and 16.7° that could be related to the slight presence of type I components in the structure. However, the intensity of these peaks denotes a predominance of CNC type II. Once SWCNTs are dispersed in CNC, the resulting XRD patterns (Fig. 1c, d) highly resemble those of the original CNC dispersants. This again suggests an excellent debundling and individualization of the starting SWCNTs caused by CNC.

In addition, the specific surface area (*S*_BET)_ and the porosity of the freeze-dried hybrids were determined. From the shape of the isotherms displayed in Fig. S2, it is visible how these spongy solids have almost no trace of micropores; they are essentially meso/macroporous with similar texture. The *V*_T_ in both cases is identical (0.13 cm^3^/g), meaning that the overall porosity does not depend on the CNC type. However, the *S*_BET_ differs from one hybrid to the other, being 54 ± 8 m^2^/g for CNC-I/SW and 41 ± 6 m^2^/g for CNC-II/SW. This is not a significant difference, exhibiting an equivalent specific surface area, but with a distinctive feature. The steepest end of the adsorption branch of CNC-II/SW at the highest relative pressure values with respect to the CNC-I/SW hybrid points to the fact that the former would be more macroporous and the latter would be more mesoporous.

### Characterization and performance of CNC/SW-based electrodes

Nanomaterial-based electrodes (CNC/SW hybrids and SWCNT) were fabricated by removing the carbon working electrode of a SPCE and then by adding the corresponding carbon nanomaterial suspension. This allows us to asseverate that the electrochemical behavior of these electrodes is only due to the carbon nanomaterials. The surface morphology of these electrodes was analyzed by SEM. As shown in Fig. S3, CNC/SW hybrids with a fibrillar structure are homogeneously dispersed across the whole substrate, forming an entangled network, ensuring a high contact probability with the analytes to be determined. In addition, the fibers are better defined for CNC/SW than for SWCNT modified electrodes, probably due to the lower presence of impurities.

**Fig. 2:**
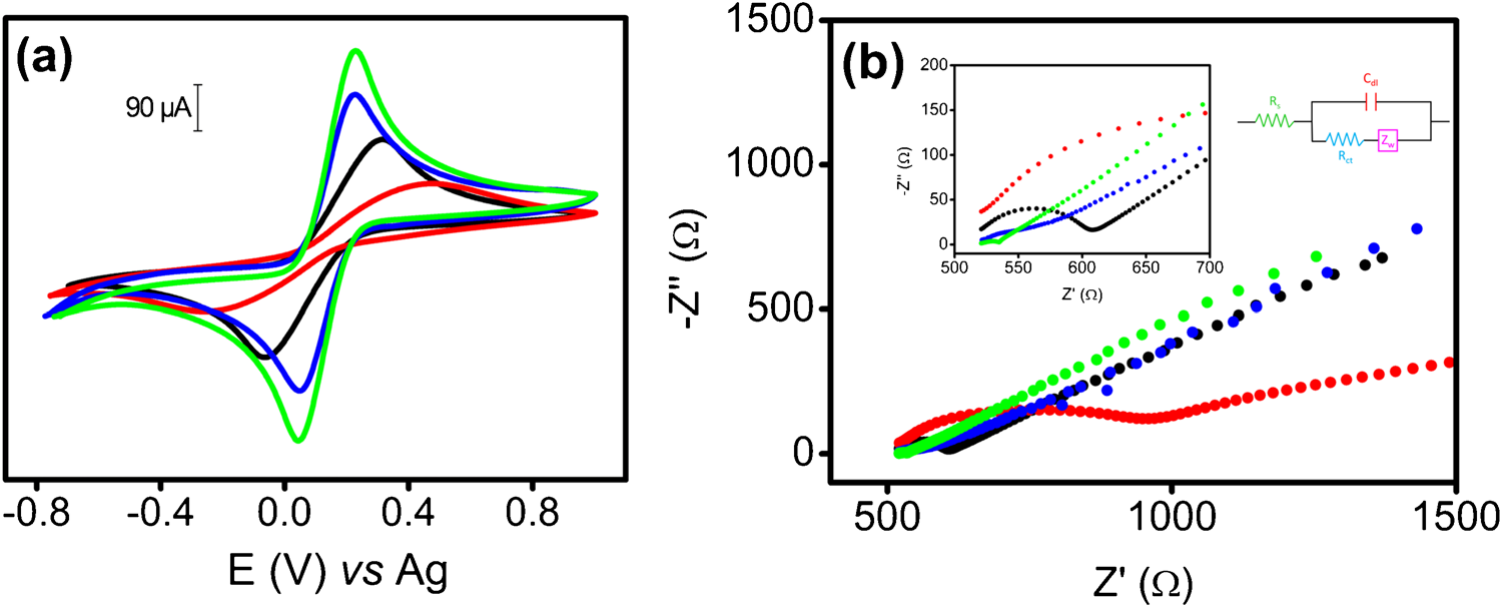
**a** CV of SPCE (black line), SWCNT-SPE (red line), CNC-I/SW (blue line), and CNC-II/SW (green line) of 5 mM Fe(CN)_6_^4−^/Fe(CN)_6_^3−^ in 0.1 M KCl. Scan rate 0.1 V s^−1^. **b** Nyquist plots corresponding to SPCE (black line), SWCNT-SPE (red line), CNC-I/SW (blue line), and CNC-II/SW (green line). Frequencies ranged from 100,000 to 0.01 Hz using 5 mM Fe(CN)_6_^4−^/Fe(CN)_6_^3−^ in 0.1 M KCl electrolyte as redox probe

The CNC/SW electrodes were also characterized by electrochemical techniques and compared to two controls (SPCE and SWCNT-SPE). Figure 2a shows cyclic voltammograms of Fe(CN)_6_^4−^/Fe(CN)_6_^3−^ (ferro/ferri system) for each material. Both CNC/SW electrodes (blue and green lines) showed better reversibility (lesser difference between the anodic peak potential and the cathodic peak potential, as follows: − 177 mV (CNC-I/SW) ≤  − 184 mV (CNC-II/SW) <  − 383 mV (SPCE) <  − 791 mV (SWCNT-SPE), which means faster electron transfer, and highest peak currents with respect to other materials. In addition, all electrodes were analyzed by EIS. Figure 2b shows the EIS results for SWCNT-SPE, SPCE, and CNC/SWs using ferro/ferri system. Based on Nyquist plots (Fig. 2b), our samples can be sorted according to the heterogeneous charge transfer resistance as follows: SWCNT-SPE > SPCE > CNC-I/SW ≥ CNC-II/SW. Based on both studies, clearly CNC/SWs showed the best electrochemical behavior. It is indeed striking, because NC is not conductive, and thus would suggest that the electrochemical reaction only happens on SWCNTs. We hypothesized that, during the synthesis of CNC/SW hybrids, SWCNTs experienced an additional purification process, by action of SWCNT adsorption on CNC, entailing the removal of carbonaceous impurities, by selective adsorption of CNC onto the CNT surface, thereby isolating them aided by the centrifugation step.

This would explain, at least in part, the improvement of the electrochemical performance of the hybrids in comparison with bare SWCNT (used as control). This improvement of the electrochemical performance of SWCNTs due to the purification process agrees with previous literature [29].

Furthermore, the higher peak currents obtained by using CNC/SW electrodes with respect to those obtained by SWCNT-SPE may be due to the higher active surface of the former.

To confirm our hypotheses, the active surface of every electrode was electrochemically measured by CV using the Randles–Sevcik equation (see SI). The surface areas of SPCE, SWCNT-SPE, CNC-I/SW, and CNC-II/SW were 0.148 ± 0.004 cm^2^, 0.127 ± 0.004 cm^2^, 0.163 ± 0.005 cm^2^, and 0.177 ± 0.009 cm^2^, respectively. The CNC/SW hybrid–based electrode showed the highest active surface, increasing it by 20% in respect to SPCE. Moreover, this difference is even higher with respect to SWCNT-SPE (40%). This means that CNC/SW dispersions generate films with more accessible points for the electrochemical probe than bare SWCNT dispersions.

Regarding heterogeneous charge transfer and electrochemical active surface, CNC/SW hybrids offered the best analytical capabilities. Then, the analytical performance of these electrodes was evaluated by detecting several analytes with a wide variety of chemical structures and interest in the clinical field.

### Evaluation of detection capabilities of CNC/SW as electrochemical transducers

**Fig. 3:**
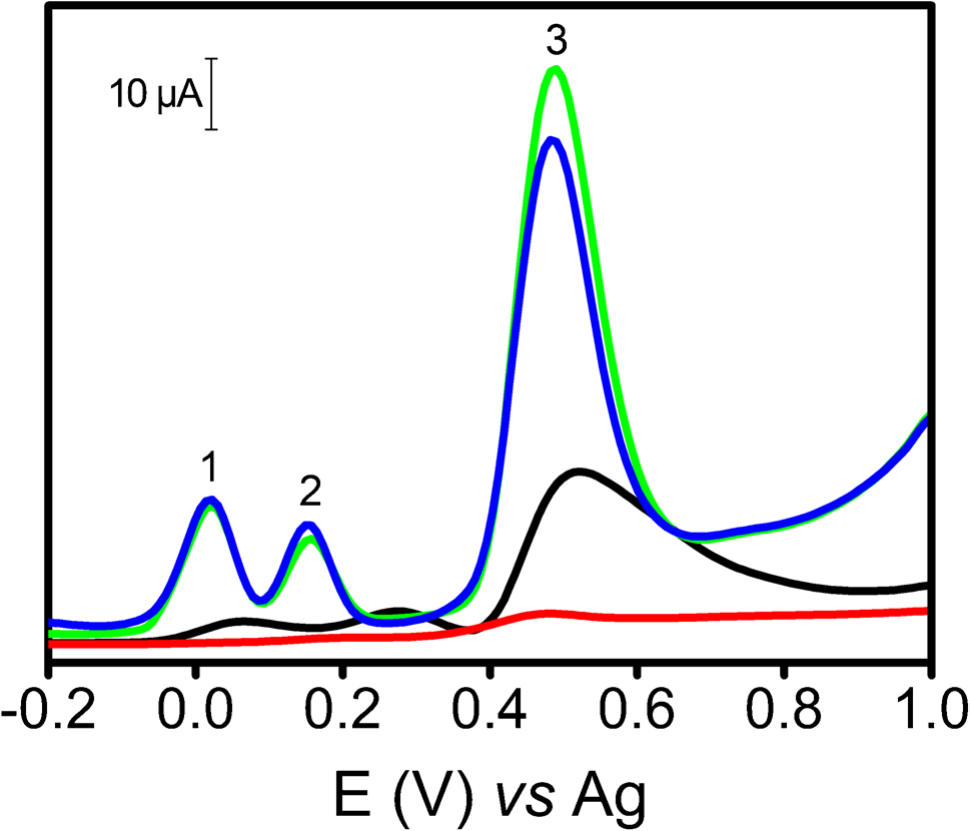
DPVs of DP 0.05 mM (peak 1), UA 0.1 mM (peak 2), and Tyr 1 mM (peak 3), using SPCE (black line), SWCNT-SPE (red line), CNC-I/SW (blue line), and CNC-II/SW (green line). Experimental conditions: 0.01 M PBS (pH = 7.4), pulse amplitude 70 mV, and scan rate 26 mV s^−1^

Target metabolites were selected for this study: dopamine (DP), uric acid (UA), and tyrosine (Tyr). All of them are involved in fundamental biological processes and their monitoring is interesting in the clinical field [30–32]. Specifically, the simultaneous detection of these compounds was carried out by DPV. As displayed in Fig. 3, CNC/SW-based electrodes yielded better electrochemical behavior in terms of sensitivity (peak intensity) and selectivity (peak resolution) than the controls (SWCNT-SPE and SPCE). On the other hand, RSD values of analyte peak heights using different electrodes (*n* = 3) were ≤ 8% for SPCE, ≤ 6% for CNC-I/SW, and ≤ 9% for CNC-II/SW.

Then, the calibration of each metabolite for the electrochemical transducers assayed (except SWCNT-SPE) was carried out. The analytical characteristics of calibration graphs are shown in the SI (Table S1). While the use of CNC/SW electrodes improves the method sensitivity (calibration slopes) in comparison to SPCE for all analytes studied (around one order of magnitude), similar LODs were obtained in all cases studied.

**Fig. 4:**
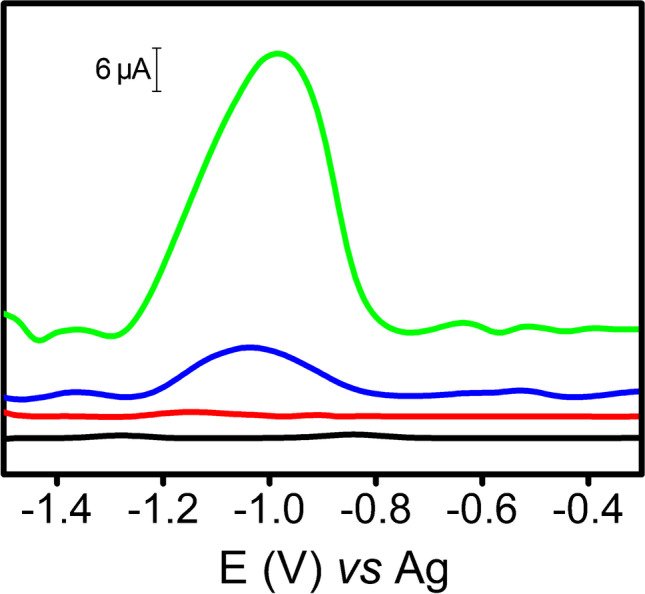
DPVs of 60 mg L^−1^ AGP-Os (VI) adduct using SPCE (black line), SWCNT-SPE (red line), CNC-I/SW (blue line), and CNC-II/SW (green line). Experimental conditions: 50 mM phosphate buffer (pH = 7.0), pulse amplitude 70 mV, and scan rate 26 mV s^−1^. Voltammograms were linearized

After studying these metabolites, we explored the potential of CNC/SW hybrids for the quantification of glycoproteins, which are biomacromolecules with high clinical relevance because some of them are used as disease biomarkers. AGP, which is an inflammatory disease biomarker [26, 33], was selected for this purpose. It was labeled with the electrochemical tag Os(VI)O_2_(OH)_2_TEMED (AGP-Os(VI) adduct) [26, 27], and then measured by DPV. Figure 4 shows the voltammograms for AGP using the aforementioned electrodes. While the use of SPCE provided a small and broad peak at − 1.3 V, CNC/SWs produced an intense and well-defined peak at − 1.0 V. More interestingly, CNC-II/SW yielded a peak higher than CNC-I/SW. Furthermore, no signal was recorded using SWCNT-SPE. In addition, the repeatability was good with RSD values for AGP peak height of 3%, 10%, and 5% for SPCE, CNC-I/SW, and CNC-II/SW, respectively (*n* = 3).

Therefore, the type of CNC resulted critical for the sensor performance in the determination of this glycoprotein of high relevance. This fact may be related to the differences in porosity. As it was, CNC-II/SW is more macroporous and CNC-I/SW is more mesoporous. This could explain why CNC-II/SW exhibits higher signal for macromolecules than CNC-I/SW, likely due to the higher amount and accessibility of its macropores for this kind of analytes [34]. Nevertheless, it is worth noting that the porosity was evaluated using freeze-drying hybrids and the electrochemical sensor operates in aqueous media, so this comparison should be taken only as indicative.

Another glycoprotein of high significance used as disease biomarker, transferrin [35], was analyzed using the same methodology (see Fig. S4). Interestingly, the same results for each kind of electrode were observed, wherein CNC-II/SW yielded the highest peak too. This finding indicates the potential significance of the cellulose polymorphism on electrochemical sensing of glycoproteins.

Due to the better electrochemical behavior of the CNC-II/SW transducer on AGP detection, it was chosen to carry out its quantitative determination. The main analytical characteristics of methodological calibration are listed in Table 2. CNC-II/SW hybrids showed a linear response in the studied AGP concentration range (from 20 to 100 mg L^−−1^), showing around 20 times higher sensitivity in terms of calibration slope (0.02 ± 0.01) than CNC-I/SW, with a LOD of 7 mg L^−1^ (3S/N criterion).

**Table 2:** Analytical characteristics of calibration graphs for AGP using CNC-II/SW

Linear range (mg L^−1^)	*r*	*a* ± *s*_a_ (µA)	*b* ± *s*_b_ (µA mg^−1^ L)	LOD (mg L^−1^)
20–100	0.998	3 ± 1	0.40 ± 0.05	7

Finally, the CNC-II/SW-based sensor accuracy was carefully evaluated. The amount of AGP was quantified in a certified reference material (human serum) by DPV using CNC-II/SW, obtaining a value of 447 ± 42 mg L^−1^ (*n* = 3). The concentration of AGP in the certified reference material is 617 mg L^−1^, so the recovery was 72 ± 7% (*n* = 3). It is necessary to bear in mind that these recovery levels were reproducible and quantitative well-enough, since the maximum recovery level obtained for AGP using acid precipitation, necessary for its selective determination, is 80% [26, 28].

This simple sample preparation methodology is also very attractive because the use of bioreagents (antibodies, enzymes, etc.) is avoided, showing a very good selectivity for AGP determination in complex samples. In addition, the increase of AGP levels during an inflammatory process is 2- to 3-folds [36], so that the sensor would be able to clearly monitor these changes.

## Conclusions

CNC/non-oxidized SWCNT hybrid transducers smartly exploited the excellent dispersive action of CNC towards non-oxidized SWCNTs in water (scarcely addressed), which is of relevance to preserve their conductive properties, avoiding the use of organic solvents or the incorporation of toxic surfactants during their processing, making the CNC/SW hybrids promising nanomaterials for electrochemical detection following greener approaches.

They showed their potential applicability in clinical sensing by detecting target metabolites and glycoproteins, with an improved analytical performance in comparison with commercial electrodes or their counterparts with just non-oxidized SWCNTs.

Interestingly, the CNC polymorphism was critical in the electrochemical detection of AGP, in which CNC-II/SW hybrid showed greater sensitivity than CNC-I/SW, probably due to its macroporous structure. However, more studies to understand the CNC polymorphism influence on electrochemical sensing of glycoproteins and other related macromolecules are highly needed.

Given the increasing interest in using greener methods to process and exploit the enormous potential of carbon nanostructures, this work opens new avenues in the development of electrochemical sensors for detecting a wide plethora of analytes.

## Supplementary Information

Below is the link to the electronic supplementary material.

Supplementary file1 (DOCX 1905 KB)

## Abbreviations

AGP: Alpha-1-acid glycoprotein
CNC: Cellulose nanocrystals
CNC/SW: Cellulose nanocrystal/single-walled carbon nanotube
CNM: Carbon nanomaterials
CNT: Carbon nanotube
CV: Cyclic voltammetry
DLS: Dynamic light scattering
DMF: *N*,*N*-Dimethylformamide
DP: Dopamine
DPV: Differential pulse voltammetry
EIS: Electrochemical impedance spectroscopy
MWCNT: Multi-walled carbon nanotube
NC: Nanocellulose
NC/CNM: Nanocellulose/carbon nanomaterials
NIR: Near infrared
PB: Phosphate buffer
PBS: Phosphate buffer saline
SEM: Scanning electron microscope
SPCE: Screen-printed carbon electrode
SWCNT: Single-walled carbon nanotube
TEMED: *N*,*N*,*N*′,*N*′-Tetramethylenediamine
Tyr: Tyrosine
UA: Uric acid
XRD: X-ray diffraction
