# Supplementary material for: Effect of nanocellulose polymorphism on electrochemical analytical performance in hybrid nanocomposites with non-oxidized single-walled carbon nanotubes
Source: Mikrochim Acta. 2022 Jan 14;189(2):62. doi: 10.1007/s00604-021-05161-w (PMC8816370; doi:10.1007/s00604-021-05161-w)
Supplement: Supplementary file 1 — Supplementary file1 (DOCX 1905 KB) [file 604_2021_5161_MOESM1_ESM.docx]

**Supplementary Information**

**EFFECT OF NANOCELLULOSE POLYMORPHISM ON ELECTROCHEMICAL ANALYTICAL PERFORMANCE IN HYBRID NANOCOMPOSITES WITH NON-OXIDIZED SINGLE-WALLED CARBON NANOTUBES**

**Silvia Dortez^1^, Tania Sierra^1^, Miguel Á. Álvarez-Sánchez^2^, José M. González-Domínguez^2^, Ana M. Benito^2^, Wolfgang K. Maser^2^, Agustín G. Crevillen^3*^, Alberto Escarpa^1,4*^**

^1^ Department of Analytical Chemistry, Physical Chemistry and Chemical Engineering, University of Alcalá, E-28871, Alcalá de Henares, Madrid, Spain.

^2^ Group of Carbon Nanostructures and Nanotechnology, Instituto de Carboquímica, ICB-CSIC, C/ Miguel Luesma Castán 4, 50018, Zaragoza, Spain.

^3^ Department of Analytical Sciences, Faculty of Sciences, Universidad Nacional de Educación a Distancia (UNED), E-28040, Madrid, Spain.

^4^ Chemical Research Institute “Andrés M. del Río” (IQAR), University of Alcalá, E-28805 Alcalá de Henares, Madrid, Spain.

* Corresponding author:

Alberto Escarpa ([alberto.escarpa@uah.es](mailto:alberto.escarpa@uah.es))

Agustín G. Crevillen ([agustingcrevillen@ccia.uned.es](mailto:agustingcrevillen@ccia.uned.es))

**Synthesis of CNC type I and II**

Synthesis of both NCs types I and II was performed according to the process described by a preceding work in our laboratories, reported elsewhere [1]. Briefly, in a spherical flask, 10 g of microcrystalline cellulose were mixed in a sonication bath (45 KHz) with ultrapure water (45 mL) for 10 min. Secondly, 45 mL of H_2_SO_4_ (98%) was added dropwise under stirring while externally cooled with an ice bath. The viscous medium obtained after the addition has a 64% concentration of acid requires vigorous stirring to avoid the cellulose carbonization. Subsequently, the flask is stirred on a hot plate under different conditions depending on the desired nanocellulose type: 70 °C for 10 minutes to obtain CNC type I, and 27 °C for 5 hours to obtain CNC type II. Then, the reaction medium is poured in cold ultrapure water (1 L) and left to settle overnight, the supernatant liquid was decanted off and the sediment was dialyzed against ultrapure water using a dialysis membrane (SpectraPor®, Spectrum Labs, regenerated cellulose, 6–8 KDa) submerged in 5 L of ultrapure water, until reaching neutral pH in the outer waters. The obtained dialyzed CNC colloid was centrifuged at 9300 rcf for 1 min to isolate CNC, which remains in suspension after centrifugation. In order to measure the average CNC concentration (in mg mL^-1^) and process yield (in wt %), three aliquots of 30.0 mL were freeze-dried and weighed.

**Characterization of CNC/SWCNTs by Visible-NIR**

Vis-NIR absorption is one of the most accurate technique to evaluate SWCNT purity in liquid dispersion. The spectral features seen in the Vis-NIR region are dependent on the SWCNTs structural aspects (such as chirality, diameter, conductive nature, etc.), and emerge as peaks or bands corresponding to specific metallic or semiconducting Van Hove transitions in the electronic density of states [2]. Besides, pristine SWCNTs samples also contain other forms of carbon, metallic particles and other kinds of impurities, causing a huge increase in the SWCNTs Vis-NIR absorption bands when these impurities are removed. Then, the analysis of the magnitude of these bands brings an estimate of the SWCNTs carbonaceous purity [3]. Since these transitions stem from the electronic configuration of the SWCNTs sidewalls, the Vis-NIR spectral features will mainly be affected by any physical or chemical process entailing a disruption of the native sp^2^ lattice (i.e. covalent functionalization) [4]. Thus, non-covalent interactions with any chemical species does not induce per se any alteration in the SWCNTs Van Hove transitions, aside from an intensification of the bands due to a purification effect if they are combined with techniques such as centrifugation. In former works, we determined that the adsorption of a given SWCNT sample with different surfactants or biopolymer dispersants did not substantially affect its Vis-NIR spectral features, as per their practically equal spectral profile and almost unchanged extinction coefficients [5].


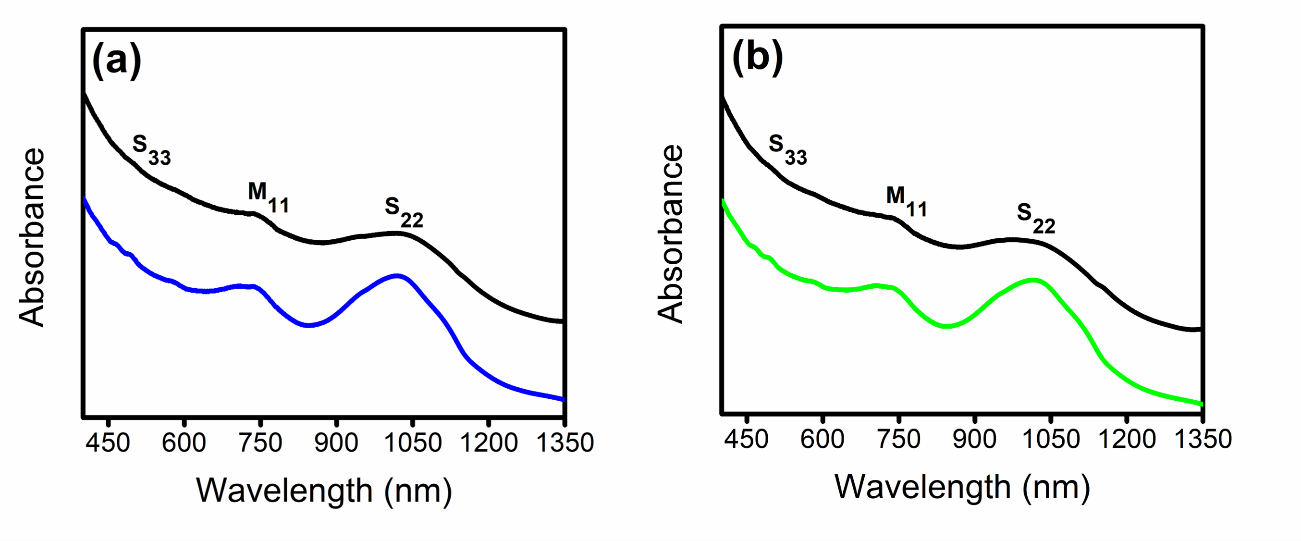


**Fig. S1** Vis-NIR absorbance plots of SWCNTs dispersed in CNC type I (**a**, in blue) and CNC type II (**b**, in green) together with the non-centrifuged counterparts (in black) and the electronic transitions associated

**Characterization of CNC/SWCNTs by BET**


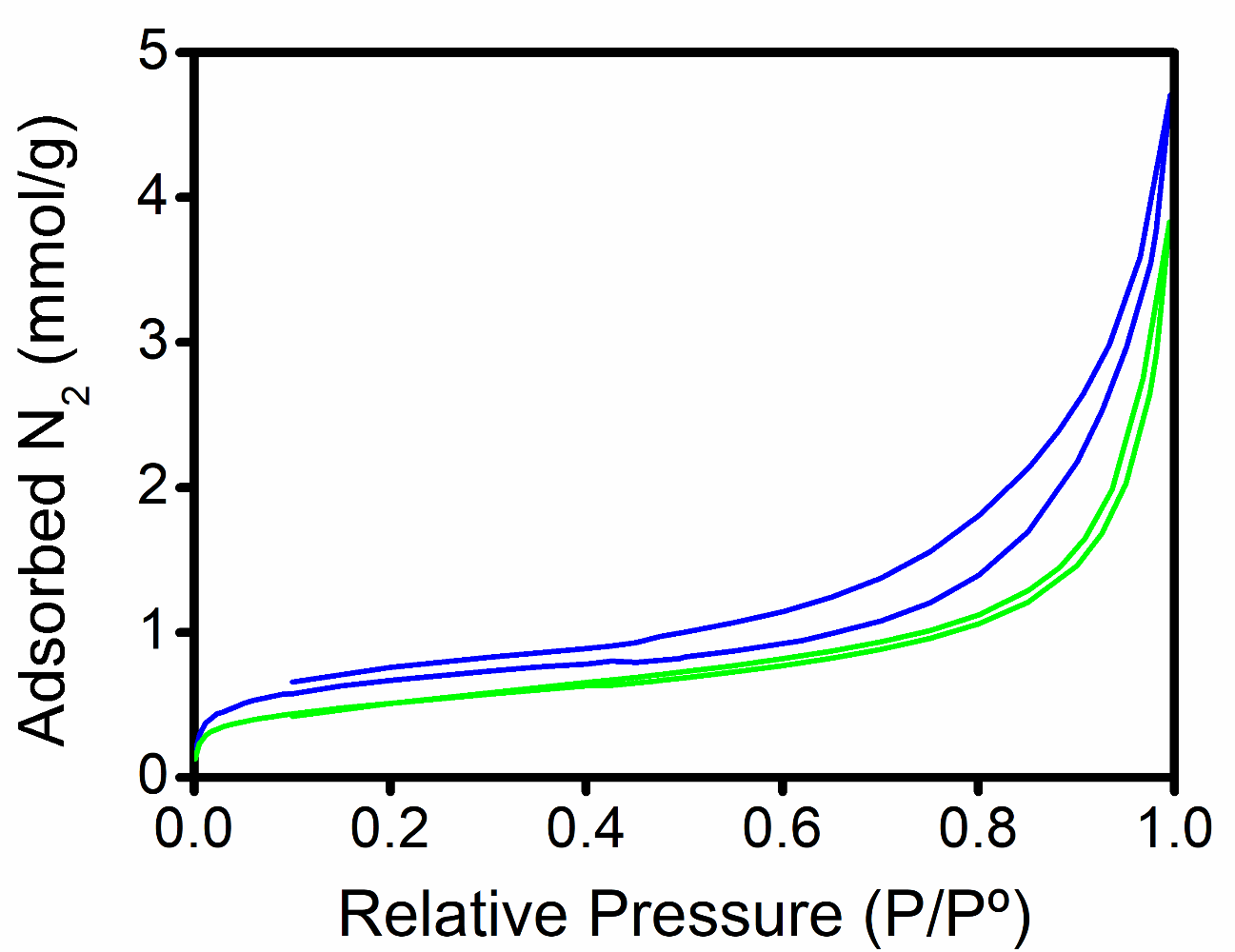


**Fig. S2** N_2_ adsorption-desorption isotherms for the CNC/SW freeze-dried hybrids: CNC-I/SW (blue plot); CNC-II/SW (green plot)

**Characterization of CNC/SWCNTs by SEM**


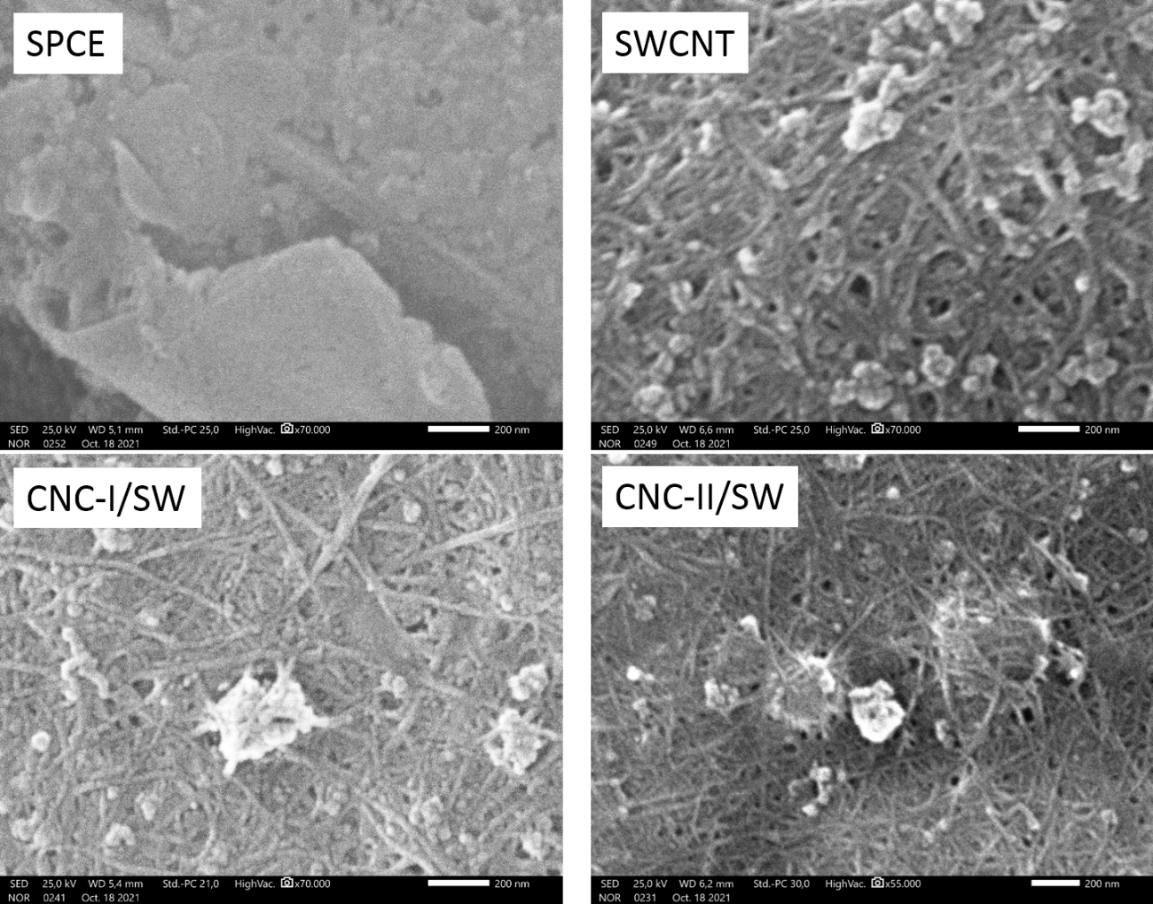


**Fig. S3** SEM characterization of SPCE and the different electrodes based on carbon nanomaterials

**Measurement of electrode surface area**

The electroactive surface area of an electrode can be measured by CV using a reversible redox probe and the Randles–Sevcik equation (at 25 °C):

$$i_{p}=2,69 x {10}^{5}n^{3/2}AD_{0}^{1/2}v^{1/2}C_{o}$$

where *i_p_* refers to the anodic peak current, *n* is the number of electrons transferred, *A* is the electrode surface area, *D_o_* is the diffusion coefficient of the redox probe, *C_o_* is the concentration of the redox probe, and *v* is the scan rate. Ru(NH_3_)_6_Cl_3_ was selected as redox probe and the electrode surface area was calculated by the slope of *i_p_* *vs* *v^1/2^* plot. With this aim, CVs of SPCE, SWCNT-SPE, CNC-I/SW and CNC-II/SW (n = 3) were recorded at different scan rates (from *v* = 0.01 to *v* = 0.1 V s^-1^) using 1 mM Ru(NH_3_)_6_Cl_3_ in 0.5 M KNO_3_ electrolyte (n = 1 and D_0_ =7.74 x 10^−6^ cm^2^ s^−1^).

**Figures of merit of calibration graphs for small metabolites**

**Table S1** Analytical characteristics of calibration graphs for dopamine, uric acid, and tyrosine

| **Metabolite** | **CNC polymorphism** | **Linear Range (µM)** | **r** | **a ± s_a_**  **(µA)** | **b ± s_b_**  **(µA µM^-1^)** | **LOD (µM)** |
| --- | --- | --- | --- | --- | --- | --- |
| **Dopamine** | SPCE | 7.5 – 75 | 0.993 | -0.015 ± 0.007 | 0.048 ± 0.003 | 0.4 |
|  | CNC-I/SW |  | 0.996 | -0.23 ± 0.07 | 0.556 ± 0.009 | 0.4 |
|  | CNC-II/SW |  | 0.998 | -1.5 ± 0.8 | 0.583 ± 0.005 | 4 |
| **Uric acid** | SPCE | 25 – 200 | 0.98 | 0.44 ± 0.01 | 0.019 ± 0.001 | 1 |
|  | CNC-I/SW |  | 0.990 | 6.6 ± 0.3 | 0.121 ± 0.006 | 9 |
|  | CNC-II/SW |  | 0.991 | 1.3 ± 0.2 | 0.18 ± 0.04 | 3 |
| **Tyrosine** | SPCE | 75 – 750 | 0.995 | 2.53 ± 0.03 | 0.023 ± 0.001 | 4 |
|  | CNC-I/SW |  | 0.996 | 5 ± 1 | 0.113 ± 0.005 | 26 |
|  | CNC-II/SW |  | 0.999 | 4 ± 1 | 0.13 ± 0.02 | 23 |

**Differential Pulse Voltammetry of Transferrin-Os(VI) aduct on CNC/SWCNTs transducers and controls**





**Fig. S4** DPVs of 1.9 g L^-1^ transferrin-Os (VI) adduct using: SPCE (black line), SWCNT-SPE (red line), CNC-I/SW (blue line) and CNC-II/SW (green line). Experimental conditions: 50 mM phosphate buffer (pH = 7.0), pulse amplitude 70 mV and scan rate 26 mV s^-1^. Voltammograms were linearized

**References**

1. González-Domínguez JM, Ansón-Casaos A, Grasa L, Abenia L, Salvador A, Colom E, Mesonero JE, García-Bordejé JE, Benito AM, Maser WK (2019) Unique Properties and Behavior of Nonmercerized Type-II Cellulose Nanocrystals as Carbon Nanotube Biocompatible Dispersants. Biomacromolecules 20:3147–3160. <https://doi.org/10.1021/acs.biomac.9b00722>
2. Hodge SA, Bayazit MK, Coleman KS. and Shaffer, Milo SPS (2012) Unweaving the rainbow: a review of the relationship between single-walled carbon nanotube molecular structures and their chemical reactivity. Chem Soc Rev 41:4409-4429. <https://doi.org/10.1039/C2CS15334C>
3. Ansón-Casaos A, González M, González-Domínguez JM, Martínez MT (2011) Influence of air oxidation on the surfactant-assisted purification of single-walled carbon nanotubes. Langmuir 27:7192–7198. <https://doi.org/10.1021/la200730k>
4. Blanch AJ, Lenehan CE, Quinton JS (2012) Dispersant effects in the selective reaction of aryl diazonium salts with single-walled carbon nanotubes in aqueous solution. J Phys Chem C 116:1709–1723. <https://doi.org/10.1021/jp208191c>
5. Ansón-Casaos A, González-Domínguez JM, Lafragüeta I, Carrodeguas JA, Martínez MT (2014) Optical absorption response of chemically modified single-walled carbon nanotubes upon ultracentrifugation in various dispersants. Carbon N Y 66:105–118. <https://doi.org/10.1016/j.carbon.2013.08.048>
